# Preoperative esophagogastroduodenoscopy findings and effects on laparoscopic Roux-en-Y gastric bypass in area with high prevalence of *Helicobacter pylori *infection: multi-center experience in Iran 

**Published:** 2022

**Authors:** Mehdi Alimadadi, Seyedali Seyedmajidi, Sina safamanesh, Elena Zanganeh, Seyed Ashkan Hosseini, Shahin Hajiebrahimi, Mohammadreza Seyyedmajidi

**Affiliations:** 1 *Golestan Research Center of Gastroenterology and Hepatology (GRCGH), Golestan University of Medical Sciences, Gorgan, Iran*; 2 *Dental Materials Research Center, Health Research Institute, Babol University of Medical Sciences, Babol, Iran*; 3 *Minimally Invasive Surgery Research Center, Tehran University of Medical Sciences, Tehran, Iran*; 4 *Mashhad University of Medical Sciences, Mashhad, Iran*

**Keywords:** Gastric bypass surgery, Esophagogastroduodenoscopy, Helicobacter pylori

## Abstract

**Aim:**

The current study aimed to evaluate EGD findings effects on laparoscopic Roux-en-Y gastric bypass (RYGB) plan and time in areas with a high prevalence of *Helicobacter pylori* infection.

**Background:**

Esophagogastroduodenoscopy (EGD) and *Helicobacter pylori *testing are routine parts of preoperative assessment of bariatric surgery at many centers

**Methods:**

This was a crosssectional study of all patients underwent EGD and histopathological examination before laparoscopic RYGB in three gastroenterology centers in Iran between January 2018 and December 2020.

**Results:**

In total, 637 patients (52.4% female) were enrolled, of which 46.8% had no abnormal mucosal appearance. In 1.7%, surgery was canceled (gastric adenocarcinoma, gastric intestinal metaplasia, GIST, and esophageal varices). The prevalence of *H. pylori* was 61.5%, and there was no statistical difference between groups of normal and abnormal EGD; however, surgery was postponed after* H. pylori* eradication in both groups. Overall, 44.4% of patients with esophagitis (any grade), peptic ulcer disease, erosive and non-erosive gastritis/duodenitis, and short segment Barret’s esophagus needed medical management. Small- or medium-sized sliding hiatal hernias were seen in 18.7% of patients with no effect on surgery. Moreover, 88.8% of patients with normal mucosal appearance were asymptomatic, but 92.6% in the group with abnormal EGD were symptomatic (*p*=0.01). Changes in surgical plan and time occurred in 63.6%, but after eliminating *H. pylori* eradication, it was 15.4%.

**Conclusion:**

Considering gastric cancer and the high prevalence of *H. pylori* in Iran, using EGD and histopathological examination as an investigation in the preoperative assessment would have a significant impact on patients undergoing RYGB surgery.

## Introduction

 Obesity has become a common health problem all over the world and its prevalence is increasing in both developed and developing countries. Obesity and overweight are the world's fifth cause of mortality, and 2.6 million people die due to this disorder annually (1). Therefore, it is probable that the most important of risk factors fulfill the criteria for being governmental health priorities. In 2016, the national prevalence rates of normal weight, obesity, and overweight/obesity in Iranian adults were estimated at 36.7%, 22.7%, and 59.3%, respectively (2). One analysis in Iran showed that 33.78%, 10.25%, and 30.56% of cases of the prevalent diabetes mellitus were attributable to overweight (Body Mass Index: BMI ≥ 25 kg/m2), general obesity (BMI ≥ 30 kg/m2), and central obesity (waist circumference ≥ 90 cm), respectively (3). 

Conservative management of obesity can only induce a nearly 10% weight loss and may provide cardiac and metabolic benefits (4). However, the American Society for Metabolic and Bariatric Surgery (ASMBS) recommended bariatric surgery for patients with a BMI ≥ 40 with or without coexisting comorbidities, and for those with a BMI ≥ 35 with severe obesityrelated medical conditions or remarkably impaired quality of life (5). The evaluation before bariatric surgery consists of history taking, physical examination, laboratory tests, psychology, endocrine, cardiopulmonary, and gastrointestinal (GI) assessments. The GI evaluation might include screening for *Helicobacter pylori* (*H. pylori) *in high prevalence areas, or if clinically indicated, and esophagogastroduodenoscopy (EGD) (5). This practice evaluated endoscopic findings, *H. pylori *status, and histopathological examination in the preoperative assessment of patients undergoing laparoscopic Roux-en-Y gastric bypass (RYGB) in Iran with a high prevalence of *H. pylori*. 

## Methods

This study was a crosssectional evaluation of all patients who underwent upper GI endoscopy before laparoscopic RYGB surgery in three gastroenterology clinics in Iran (Gorgan, Tehran, and Mashhad) between January 2018 and December 2020 (3 years). EGDs were done and two specimens were obtained from the antrum to assess *Helicobacter pylori *status and histopathological examination. All participants signed an informed consent form. History taking, physical examination. and patients’ data collected included age, gender, BMI, *H. pylori *status, EGD and histopathological results as outcome variables. 

All data was analyzed using Statistical Package for Social Sciences Ver.20 (SPSS, IBM Corp.; Armonk, NY, USA). The values were expressed as mean ± standard deviation (SD) for continuous variables and percentages for categorical variables. The normality of distribution of data was assessed using the Kolmogorov-Smirnov test. 

## Results

**Table 1:** Patients’ endoscopic findings on the basis of sex, BMI

Endoscopic findings	Sex			BMI			Total
	male	female	*p*. value	35≤BMI<40	40≤BMI	*p*. value	
Normal mucosal appearance	148	191	0.20	161	178	0.35	339
Esophagitis (LA* A & B)	30	26	0.59	27	29	0.78	56
Esophagitis (LA C & D)	1	2	0.56	1	2	0.56	3
Barret’s esophagus	4	3	0.70	3	4	0.70	7
Esophageal varices	1	1	-	0	2	-	2
Non-erosive gastritis / duodenitis	74	70	0.73	79	65	0.24	144
Erosive gastritis/ duodenitis	22	18	0.52	19	21	0.75	40
Gastric / duodenal ulcers	19	14	0.38	18	15	0.60	33
Gastric polyps	4	5	0.73	5	4	0.73	9
Gastric bezoar	1	1	-	1	1	-	2
Gastric adenocarcinoma	1	0	-	1	0	-	1
Gastrointestinal Stromal Tumor (GIST)	0	1	-	1	0	-	1
Total	305	332	0.28	316	321	0.843	637

* Los Angeles classification

**Table 2:** Correlation between *H. pylori* status and endoscopic findings

*H. pylori* status	Normal mucosal appearance	Abnormal mucosal appearance	Total
Positive	208 (61.4%)	184 (61.7%)	392 (61.5%)
Negative	131 (38.6%)	114 (38.3%)	245 (38.5%)
Total	339	298	637

**Table 3:** Correlation between upper GI symptoms & signs and endoscopic findings

Upper GI symptoms & signs	Normal mucosal appearance	Abnormal mucosal appearance	Total
Positive	38 (11.2%)	276 (92.6%)	314 (49.3%)
Negative	301 (88.8%)	22 (7.4%)	323 (50.7%)
Total	339	298	637

A total of 637 patients, of whom 332 (52.4%) were female, were referred to the endoscopy units for preoperative EGD before laparoscopic RYGB surgery. Participants’ age ranged between 19 and 52 years with a mean ± SD of 34.4 ± 9.2 years. The average BMI and SD of patients was 45.1 ± 7.3 kg/m2. Patients’ endoscopic findings on the basis of sex and BMI are displayed in Table 1. There were no statistical differences in sex and BMI between groups of EGD findings.

In 119 EGDs (18.7%), small- or medium-sized sliding hiatal hernias were seen without effect on surgery plan or time. The pathology reports (Table 2) showed that the prevalence of *H. pylori *in patients with normal EGD, abnormal mucosal appearance, and in total cases was 61.4%, 61.7%, and 61.5%, respectively (*p*=0.74). Surgery was postponed after *H. pylori *eradication.

Without considering *H. pylori* status and sliding hiatal hernia, the patients were categorized into four groups according to the endoscopic and histopathologic results:

**Normal EGD**

In all, 53.2% of patients had no abnormalities in EGD and histopathological examination.

**Minor abnormal findings**

On the basis of Los Angeles (LA) classification, esophagitis LA (A & B) and non-erosive gastritis / duodenitis were seen in 8.8% and 22.6%, respectively. Seven individuals had short segment Barret’s esophagus without dysplasia that did not change the plan of surgery. Nine patients had few tiny polyps in stomach all of which were hyperplastic in pathology and removed totally by EGD before surgery. Gastric phytobezoars were seen in 2 cases and were successfully removed with endoscopic intervention and before surgery. Gastric motility disorders were ruled out with gastric emptying scintigraphy.

**Major abnormal findings**

Esophagitis LA (C & D), erosive gastritis/ duodenitis and peptic ulcers were seen in 0.4%, 6.3%, and 5.2%, respectively. In these patients, surgery was postponed at least 3 months to complete treatment and repeat EGD.

**Findings that were considered contraindications for surgery**

Eleven patients (1.7%) in the current sample were found to have pathologies that were considered contraindications for surgery. Two patients had esophageal varices, one had malignant ulcer in the lesser curvature of the gastric body, and one had gastrointestinal stromal tumor (GIST) in the gastric fundus. In seven patients, histopathological examination of the antrum showed intestinal metaplasia without dysplasia; therefore, surgery was cancelled for surveillance EGD.

In Table 3, 88.8% of patients with normal mucosal appearance in EGD were found to be asymptomatic, but only 7.4% in the group with abnormal EGD (*p*=0.01) had this result. Changes in surgical plan and time occurred in 63.6% cases, but only in 15.4% after eliminating *H. pylori* status.

## Discussion

In the past, European guidelines recommended routine EGD in the preoperative assessment of bariatric surgery, while North American guidelines recommended a selective approach (6, 7). Now, however, clinical practice guidelines of the European Association for Endoscopic Surgery (EAES) on bariatric surgery (update 2020) have no recommendation for routine *H. pylori *eradication and conditional recommendation for EGD as a diagnostic test prior to bariatric surgery on the basis of available evidence. This panel provided that selective endoscopy in patients with upper abdominal symptoms might be more appropriate (8).

One meta-analysis that included 12,261 patients showed that the proportion of EGD findings in changing surgical management was only 7.8%, and after discarding benign findings, this was 0.4%. Changing in medical management was 27.5%, but after eliminating *H. pylori *eradication, this was 2.5% (6). On this topic, two more systematic reviews were available. The first one including 6845 patients, and suggested changes in surgical management after EGD in 7.8% included delays in surgery due to gastritis or peptic ulcer disease, major changes in the planned procedure, and additional EGD for suspicious lesions. The second meta-analysis of 20 studies on 5140 patients found a management change in 27.5% after EGD. Changes in medical management included *H. pylori *eradication and initiation of proton-pump inhibitors or histamine blockers (9, 10).

The main reason for *H. pylori* screening in patients undergoing bariatric surgery was to minimize postoperative complications such as marginal ulcers and viscus perforation (11, 12). The multivariable analysis of one cohort found *H. pylori* status to be the most important independent predictor of marginal ulceration in patients undergoing RYGB, but it had little impact on the outcome of bariatric operations (13). Two other meta-analyses reported that the odds for marginal ulcer and postoperative complications after bariatric surgery were similar for *H. pylori*-positive versus *H. pylori*-negative patients. Similarly, there was no firm evidence on postoperative bleeding or leakage (14, 15). This is reflected in a conditional recommendation for routine* H. pylori* eradication (8). 

In this survey, a consecutive 637 patients who referred to the endoscopy units for preoperative EGD were analyzed. Similar to many previous studies (7, 16, 17, 18), the number of patients who had any abnormal mucosal appearance was 46.8%. 

Gastric cancer (GC) is one of the most common cancers in Iran. According to a recent systematic review (19), the prevalence of GC in Iran is between 0.2 and 100 per 100,000 with the death rate per 100,000 people ranging from 10.6 to 15.72. The incidence of GC in patients with *H. pylori* infection was 18 times higher than other populations. Low economic level and food insecurity increased the odds of GC. In 1.7% of cases in the current study, surgery was canceled due to gastric adenocarcinoma, gastric intestinal metaplasia, GIST, and esophageal varices.

The prevalence of *H. pylori* in the current study was about 61.5%, and there was no statistical difference in prevalence between groups of normal and abnormal EGD findings, but surgery was postponed after* H. pylori* eradication in both groups. Overall, 283 patients (44.4%) with esophagitis (any grade), peptic ulcer disease, erosive and non-erosive gastritis/ duodenitis, and short segment Barret’s esophagus needed treatment with proton pomp inhibitors or histamine-2 receptor antagonists. Eleven patients (1.7%) needed endoscopic intervention (gastric polyps and bezoars). Small- or medium-sized sliding hiatal hernias were seen in 18.7% without effect on surgery. 

Many authors have documented a lack of correlation between patients’ symptoms and endoscopic abnormalities (16), but herein, it was found that 88.8% of patients with normal mucosal appearance in EGD were asymptomatic, and 92.6% in the group with abnormal EGD were symptomatic (*p*=0.01). Changes in surgical plan and time was 63.6%, but after eliminating *H. pylori* eradication, EGD influenced surgery time in 13.7% and surgery cancellation in 1.7% of cases. 

In conclusion, considering gastric cancer and the high estimates of *H. pylori* prevalence in Iran, using EGD and histopathological examination as investigations in the preoperative assessment would have a significant impact on patients undergoing RYGB surgery. Particularly, if clinically symptoms and/or signs are present, the patient should go through an appropriate evaluation.

## Conflict of interests

The authors declare that they have no conflict of interest.
